# Interval Timing in Children: Effects of Auditory and Visual Pacing Stimuli and Relationships with Reading and Attention Variables

**DOI:** 10.1371/journal.pone.0042820

**Published:** 2012-08-10

**Authors:** Emma E. Birkett, Joel B. Talcott

**Affiliations:** Aston Brain Centre, School of Life and Health Sciences, Aston University, Birmingham, United Kingdom; Indian Institute of Toxicology Reserach, India

## Abstract

Motor timing tasks have been employed in studies of neurodevelopmental disorders such as developmental dyslexia and ADHD, where they provide an index of temporal processing ability. Investigations of these disorders have used different stimulus parameters within the motor timing tasks that are likely to affect performance measures. Here we assessed the effect of auditory and visual pacing stimuli on synchronised motor timing performance and its relationship with cognitive and behavioural predictors that are commonly used in the diagnosis of these highly prevalent developmental disorders. Twenty-one children (mean age 9.6 years) completed a finger tapping task in two stimulus conditions, together with additional psychometric measures. As anticipated, synchronisation to the beat (ISI 329 ms) was less accurate in the visually paced condition. Decomposition of timing variance indicated that this effect resulted from differences in the way that visual and auditory paced tasks are processed by central timekeeping and associated peripheral implementation systems. The ability to utilise an efficient processing strategy on the visual task correlated with both reading and sustained attention skills. Dissociations between these patterns of relationship across task modality suggest that not all timing tasks are equivalent.

## Introduction

Motor timing in participants with developmental dyslexia and Attention Deficit Hyperactivity Disorder (ADHD) has been investigated with behavioural tasks that require the production of motor responses in synchrony with external pacing stimuli [1], [2]. Dyslexia and ADHD are both associated with subtle performance differences in the accuracy and precision of such responses compared to controls [3]. Convergent streams of evidence for perceptual differences in the detection and discrimination of the temporal parameters of auditory, visual and sensory-motor stimuli [4], [2] support the hypothesis that poor neural timing is a candidate neuro-cognitive endophenotype of dyslexia [5] and ADHD [2].

ADHD and dyslexia are developmental disorders that are typically identified in childhood. They co-occur at such a high rate (>25%) [6], [7] that they almost certainly share underlying cognitive, neurological and genetic risk factors [8], [9]. At a neurophysiological level, a risk factor related to timing functions might explain a wide array of the cognitive and behavioural symptoms, both those that are disorder-specific and those that overlap between disorder phenotypes. Although ADHD and dyslexia are defined by independent sets of behavioural symptoms, recent evidence promotes a dimensional rather than a categorical view of developmental disorders and their associated symptoms [10]. This perspective accounts for the overlap between both the diagnostic prevalence and symptoms of these disorders and highlights evidence that common underlying mechanisms likely mediate general population variability on the constructs upon which clinical diagnoses are made (i.e., reading and attention variables) [9].

One target mechanism of impairment identified for both ADHD and dyslexia is in temporal processing [2], [5], a construct that includes the ability to segregate and process incoming sequences of stimuli (i.e., rate of perception) [11]–[13] and the detection of the temporal structure of individual stimuli (i.e., perception of rate) [14], [15]. Temporal processing has been measured using an array of neurophysiological and behavioural tasks to estimate detection or discrimination thresholds for rapidly occurring stimuli or those with properties that change in real time. Here we focus on tasks involving motor timing to a periodic sensory event, paradigms that have been employed previously in clinical investigations within the field of learning disabilities [1], [3], [16]–[18] and in other populations [19]–[21].

Tasks of motor timing assess the ability to synchronise movements (typically finger movements) with external pacing stimuli. Such tasks are particularly well-suited for use with children because they allow behavioural assessment of a processing dimension where the accuracy and precision of the response requires temporal processing but not complex subjective judgements about the nature of the stimuli presented. Differences in the ability to perform motor timing tasks between clinical and control groups have been demonstrated for adults, adolescents and children with a history of developmental dyslexia [1], [14], [17], [22], with greater response variability typically demonstrated in the group with dyslexia. Such motor timing skills appear to be sensitive to individual variation in the symptom dimensions relevant to dyslexia diagnosis, such as in reading accuracy and working memory, for both clinical and control samples [1], [23], [24]. Differences in motor timing have also been reported for ADHD, where performance of clinically-derived, paediatric samples is characterised by greater response variability at the individual level [3], [25]–[27] and difficulties compared to controls in selecting the appropriate response rate [16], [25]. Such studies have also shown that motor timing differences can correlate with continuous measures of ADHD symptoms in both clinical and control samples [25], [28].

Using motor responses as behavioural indices of temporal processing has an additional advantage over other measures of this construct because response variability can be decomposed into variance components that reflect putative underlying mechanisms [29], [30]. For example, the Wing and Kristofferson [29], [31] model proposes that two main sources of variance contribute to individual behavioural responses on motor timing tasks: a timekeeping system that monitors the pacing stimuli and generates signals at appropriate intervals, and the peripheral implementation system that produces the motor response based on input from the timekeeper. The Wing and Kristofferson model has been used to investigate the components of timing difficulties in clinical populations, including those with Parkinson’s disease and patients with cerebellar lesions [19], [32]–[34]. As applied here to children with varying abilities in reading and attention, this model may highlight the independent and shared components of timing differences associated with these core symptomatic features of dyslexia and ADHD. If the temporal processing differences found in these developmental disorders have a shared causal mechanism, we expect that the efficiency of the timekeeper mechanism will be associated with both literacy and attention variables, even within a typically developing population sample. In contrast, if the shared behavioural difficulties result from different mechanisms, these variables should not share common associations with these skills within the same participants.

Given the evidence of temporal processing deficits in both ADHD and dyslexia [5], [35], [36], it is tempting to speculate that such a generic functional property of the nervous system may help to explain the high co-morbidity between dyslexia and ADHD. Such a hypothesis has strong face validity. However, studies of groups with ADHD and dyslexia have differed with respect to the sensory modality through which pacing stimuli are delivered: auditory stimuli have been typically employed in investigations of dyslexia [1], [14], [23], [37] but visual or combined auditory-visual stimuli have been predominantly used in studies of ADHD [3], [16], [26], [27]. Investigations of performance under different task parameters show that the high temporal acuity of the auditory system facilitates precise synchronisation of motor behaviour with acoustically-presented pacing stimuli [38]–[40]. In contrast, motor synchronisation to visual stimuli typically results in greater response-variability [40]–[43]. This effect has been interpreted as evidence that limited information is available to timekeeping systems in such tasks and prevents effective monitoring and updating of associated output responses [41]. Recent evidence from behavioural [44] and neuroimaging studies [42] further highlights the importance of stimulus mode as a critical variable in understanding intra- and inter-subject differences in motor synchronisation tasks.

To evaluate the potential clinical relevance of motor synchronisation tasks in the context of these important methodological considerations, we examined the behavioural effects of altering task parameters on the timing performance of children. Performance was analysed using the variance model described by Wing and Kristofferson [31]. In addition to comparisons between tasks, we assessed statistical relationships between timing variables and measures of literacy and attention which tap the key cognitive dimensions that form the core deficits in developmental dyslexia and ADHD respectively. Both of these analyses allow the validity of the task parameters to be assessed, and are important pre-requisites to the application of these methods to clinical samples, including children with developmental disorders.

## Methods

### Participants

We recruited a group of 25 children from a single primary school classroom. Participants provided informed consent under a protocol approved by the Aston University Institutional Review Board. The research was conducted according to the Declaration of Helsinki. The head teacher at the school provided written informed consent for the study to be carried out in the school and parents were sent letters with an opt-out form to be returned if they did not consent to their child taking part. Following this, the purpose of the study was explained to each child and throughout the study they were continually assessed for their willingness to participate.

Four data sets met the exclusionary criteria for the study: one child had an existing diagnosis of an emotional-behavioural disorder; one had English as a second language and two failed to complete the experimental protocol. The remaining group of 21 children comprised 10 boys and 11 girls (age range 98–127 months; three left-handed). All 21 children had received musical instruction through either home- or school-based music lessons and all had received weekly classroom-based Samba drumming lessons throughout the previous academic year.

### Psychometric Measures

The psychometric measures employed in this study assessed cognitive dimensions that are used in deriving diagnoses of developmental dyslexia and ADHD. As applied to population samples, these measures were used to determine the statistical relationships between motor timing performance and reading and attention variables across the normal range.

#### Verbal and non-verbal reasoning

The Similarities (verbal) and Matrices (non-verbal) subscales from the Wechsler Abbreviated Scale of Intelligence (WASI) [45] were administered to all participants. Age-referenced, standard scores were derived for each child using published norms.

#### Literacy

The Reading and Spelling subtests from the Wechsler Individual Achievement Test-II UK (WIAT) [46] were completed by all participants. These untimed measures assess accuracy for items graded in difficulty, from which standard scores were obtained for each child.

#### Attention measures

We obtained teacher ratings of ADHD symptoms using the ADHD Behaviour Rating Scale-Teacher Form [47], a questionnaire with separate sets of items for the assessment of inattention (ADHD-IA) and hyperactivity-impulsivity (ADHD-HI). Both subscales capture the behavioural dimensions associated with ADHD established by the Diagnostic and Statistical Manual of Mental Disorders-IV [48].

The age-standardised Same World, Opposite World task from the Test of Everyday Attention for Children (TEA-Ch) was used to assess attentional control [49]. In each task, two practice trials were followed by two test trials. The time taken to complete the Same World and Opposite World trials was recorded. The score used in statistical analyses was the percentage increase in completion time between the two tasks.

The Score! Subscale from the TEA-Ch battery [49], was also administered to the children. Performance data for each child on this measure of sustained attention was converted to a standard score using age- appropriate norms.

### Motor Timing Measures

#### Simple reaction time

All participants completed a measure of simple reaction time in response to the same individual stimuli as those used in the two modes (auditory and visual) of the finger tapping paradigm (see below). This control measure assessed the speed of simple motor responses. Participants were instructed to respond as quickly as possible to the presentation of a single stimulus with a finger-press. These responses were registered using a flat switch plate, designed to minimise vertical travel when depressed. The plate was contained within a box to prevent participants from viewing their hand whilst making responses, reducing the visual feedback available to participants. Participants responded to 10 reaction time trials in each stimulus modality and the mean reaction time and standard deviation (SD) were calculated for each condition.

#### Synchronised finger tapping

**Figure 1:**
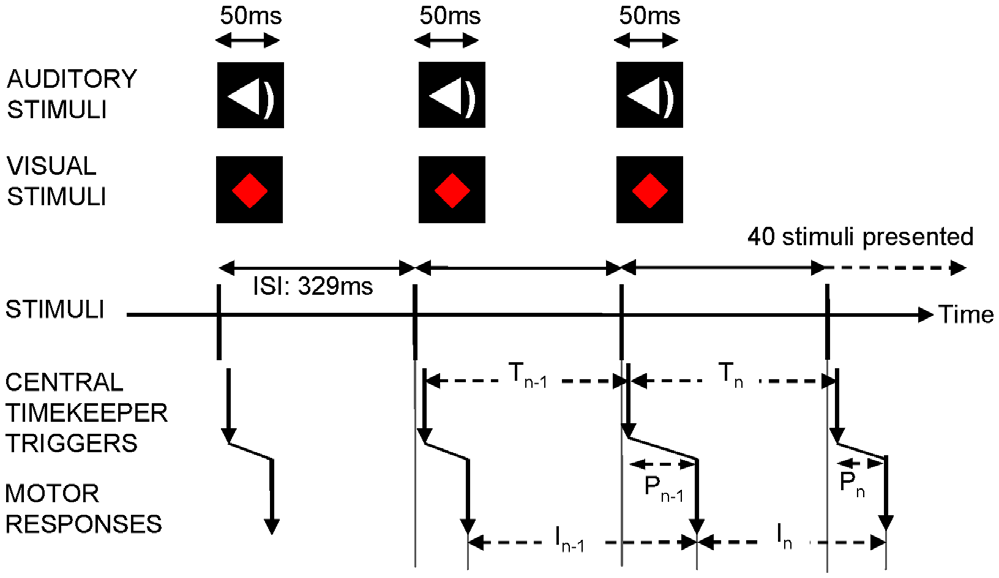
Representation of finger tapping stimuli, response synchrony and variance components. Stimulus intervals of 329 ms are represented by the lines with bidirectional arrows. A central timekeeping mechanism generates response triggers with intervals T_n_ which are subject to peripheral implementation delays (P_n_) and result in the recorded inter-response intervals of motor responses (I_n_). The mean difference from the absolute interval is calculated as inter-stimulus interval (ISI) minus I_n_.

The primary experimental measure in the study was a synchronisation task in which participants were instructed to tap their index finger of their dominant hand ‘in time’ with the repeated onsets of externally delivered pacing stimuli. The trials were presented in separate blocks, distinguished only by the different modes of stimulus presentation (auditory or visual). Within each block, participants completed three separate trial sequences, each consisting of 40 isochronous pacing stimuli with onsets timed to achieve an inter-stimulus interval (ISI) of 329 ms (see Figure 1). This tapping rate is comparable to that used previously in studies of motor timing [16], [28], [33], [34]. Responses were registered with the same switch plate described above.

In the auditory condition the stimuli were 47 ms auditory tones presented through computer speakers. The visual stimuli comprised a 2 cm diameter red diamond, presented in the centre of a CRT computer monitor (Dell Trinitron P1130) with a refresh rate of 85 Hz (11.8 frames/sec). Stimuli were presented via E-Prime presentation software [50]. Each constituent stimulus slide was timed to ensure that its offset was synchronised with the end of the fourth refresh cycle (i.e., 47 ms after stimulus presentation). At the end of this frame, the stimulus slide was replaced with a blank screen of 282 ms duration (24 frames). The timing of the stimulus and blank slide ensured that the interval between the onset of two successive stimuli was fixed at 376 ms (32 frames). Although the decay characteristics of monitor phosphors made it likely that the duration of the visual stimuli were notionally less than the 47 ms stimulus duration [51], [52], our method for timing stimuli ensured the reliability of the crucial component of the timing task, namely the consistency of the onset to onset interval.

Two further blocks of filler sequences were interspersed between trial runs to reduce potential effects related to entrainment of the stimulus presentation rate. Auditory and visual distracter blocks were comprised of three synchronisation trials each, with 20 pacing stimuli presented at an ISI of 517 ms. The order of the 4 blocks (2 speeds×2 modalities) was randomised for each child.

The tasks in the test battery were presented in a fixed order, and divided across two or three testing sessions, each of which lasted approximately 20 minutes.

### Data Analysis

The first five finger tap responses from every trial run were removed from analyses to account for stabilisation of responses. An inter-response interval (IRI) was calculated for each of the 30 remaining responses in each trial. IRIs that that were outside the range of 50% of the target interval (i.e., greater than 495 ms or less than 165 ms against the 329 ms target interval) were removed from the analysis as invalid responses on the basis that they likely resulted from response errors (for e.g., doubled responses). Data were not analysed for a given trial if more than 10 responses were deemed invalid (9.5% of total trials in the dataset). Mean and standard deviation (SD) of IRIs were calculated for each trial. The mean difference from the absolute interval was also obtained and defined as the difference between the target ISI and the IRI achieved by the participant, averaged within a trial.

From the raw data collected for each trial, estimates of the different components of timing variance were calculated using a method consistent with that described by Wing and Kristofferson [31]. This approach to the analysis of timing data assumes that for any stimulus interval, the corresponding inter-response interval (IRI) is the sum of the interval generated by a timekeeper mechanism (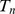) and any delays in implementation of the response from the periphery (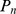), including both those delays resulting from the motor responses at the beginning (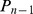) or end of that interval (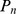). These two components are assumed to be independent, random variables, so the duration of any response interval (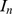) can be represented by Equation 1.This is also illustrated in Figure 1.

Equation 1: 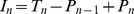


Equation 2: 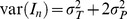


Equation 3: 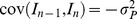


Equation 4: 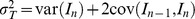


Equation 5: 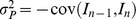


Equation 6: 
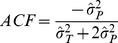


The components described in the model cannot be observed directly from the participant’s IRIs. However, the statistical dependences within human motor timing performance, detailed by Wing & Kristofferson [29], [31], provide quantifiable parameters of the system and allow estimation of the variance in these components. Variance in motor timing performance is more heavily influenced by variance in the implementation system (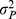) than that in the timekeeper system (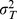), denoted by Equation 2. Each implementation delay has a differential effect on two adjacent intervals (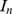 and 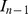), resulting in statistical dependence between these intervals and a negative correlation between them (Equation 3). The model only accounts for dependencies between adjacent intervals, and therefore assumes that non-adjacent IRIs are independent. Rearrangement of these equations allows estimation of the variance attributable to the timekeeper and implementation systems from the observed data. Timekeeper variance is estimated as the sum of the variance of the intervals plus twice the covariance of successive intervals (Equation 4). Implementation variance is estimated as the negative covariance across successive intervals (Equation 5).

Consistent with the method introduced by Kooistra et al [20], we implemented additional terms in the model. First, a drift parameter was added to the terms in Equation 1 to account for any linear trends over successive intervals. In addition, the implementation and timekeeper components were calculated based on the actual number of taps, to account for the fact that variance estimates result from a limited set of possible population samples rather than an unbiased independent sample, as assumed by Equations 2 and 3. Finally, any estimates of timekeeper or implementation variance with negative values were corrected to zero. Negative estimates of variance are theoretically impossible. Statistically, however, they are not unexpected because the tail of the sampling distribution of variance estimates can fall below zero, despite predictions of positive variance by the model [20], [53]. Such negative variance estimates do not necessarily signify a poor fit of the variance model to the data. The strategy of zero truncation has been shown to be an adequate method to account for such estimates [20], [53]. The resulting corrected estimates of timekeeper (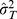) and implementation variance (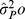) were used in subsequent analyses. The full derivation of these adjusted parameters are explained detail in Kooistra et al [20].

We also obtained the lag one auto-correlation function (ACF) from the data. This is defined as the covariance of successive intervals divided by the variance of intervals as shown in Equation 6. The ACF provides a ratio of the two variance components, in the absence of individual differences in variance magnitude, where larger values indicate a greater proportion of timekeeper variance relative to implementation variance.

## Results

### Psychometric, Literacy and Attention Measures

Descriptive statistics obtained for the psychometric measures and reaction time task are provided in Table 1.

**Table 1:** Descriptive statistics for the group of 21 children.

Measure	Mean	SD
Age (months)	115.0	9.2
Verbal Reasoning (SS)	119.4	9.4
Non-verbal Reasoning (SS)	104.9	10.5
Reading (SS)	106.9	10.2
Spelling (SS)	104.9	12.3
ADHD-IA Rating	4.8	5.4
ADHD-HI Rating	2.9	4.1
Attentional Control % increase in time	32.4	18.5
Sustained Attention (SS)	96.9	15.5
Auditory Reaction Time (ms)	335.5	81.7
Visual Reaction Time (ms)	320.2	44.7

SS: standard score (mean = 100, SD = 15), ADHD-IA: Inattention subscale score, ADHD-HI: Hyperactivity-Impulsivity subscale score.

### Motor Timing Measures

#### Stimulus modality in simple reaction time

A paired samples t-test showed that there was no significant difference in simple reaction time to auditory stimuli compared to visual stimuli (t_(20)_ = 1.12, n.s.).

#### Stimulus modality in synchronised finger tapping

Mixed-factors analyses of variance were conducted to assess motor timing performance across the within-subjects factors of mode (auditory, visual) and trial number (one, two and three). Trial number was included to assess the influence of practice effects that may arise in motor timing tasks [41].

**Figure 2:**
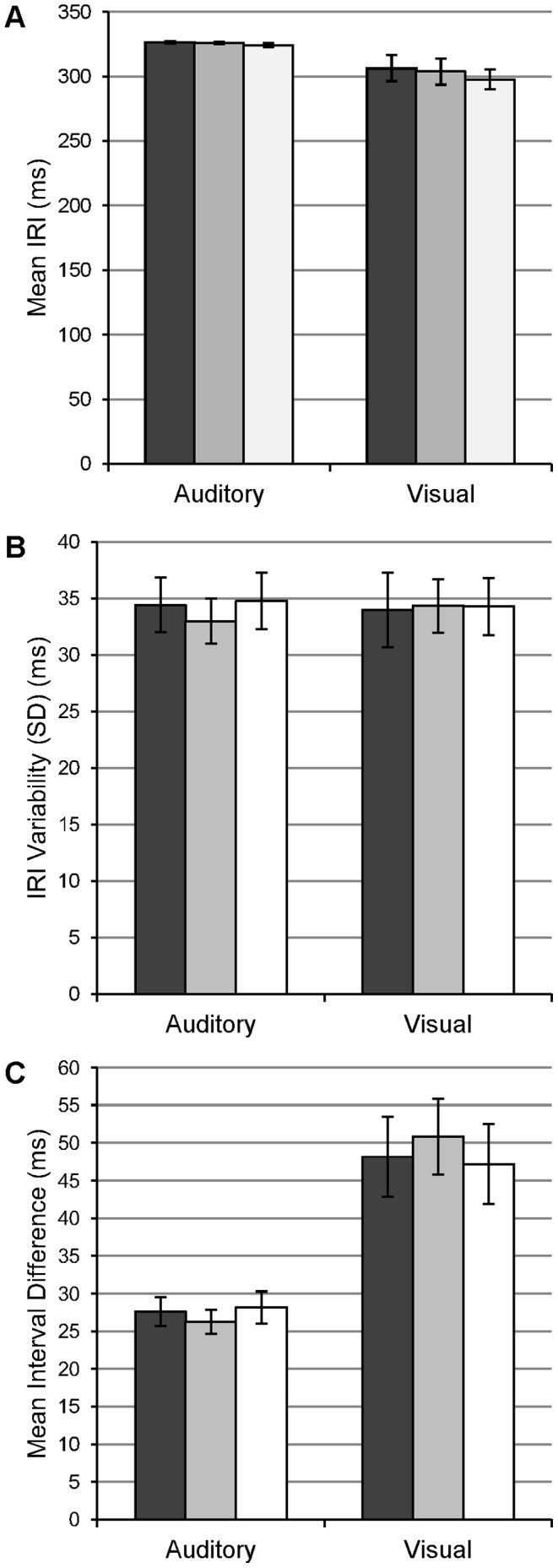
Effect of stimulus modality on finger tapping accuracy. Behavioural data across the three trials (bars: trial 1 - dark grey, trial 2 - light grey, trial 3 - mid grey)) within the two stimulus modalities. Data presented are (A) mean IRI, (B) IRI variability and (C) mean interval difference. Error bars show SEM.

Performance measures across the stimulus modes are shown in Figure 2. The effect of modality on mean IRI was marginally significant with slightly larger intervals produced in the auditory condition (mean 325 ms, S.E. 0.69) compared to the visual condition (mean 303 ms, S.E. 5.50; F_(1,12)_ = 3.56, p = 0.08, η^2^ = 0.23). In addition, there was a mean effect of trial (F_(2,24)_ = 3.69, p<0.05, η^2^ = 0.22). Average IRIs were smaller in the third trial (mean 309 ms), compared to 317 ms and 315 ms in trials 1 and 2, but the means of these conditions were not significant in pairwise post hoc tests.

IRI variability did not significantly differ across the stimulus modalities and there was no effect of trial. Additional interaction effects were not statistically significant. For the mean differences from the absolute interval, the main effect of stimulus modality was significant (F_(1,12)_ = 21.03, p<0.01, η^2^ = 0.64), with greater asynchrony between IRI and ISI demonstrated under visual conditions (mean = 49 ms, S.E. 3.03) compared to that in auditory conditions (mean = 27 ms, S.E. 1.06). Trial number did not have a significant effect on the absolute interval difference nor were there any significant interaction effects.

#### Stimulus modality and decomposed timing variance

**Figure 3:**
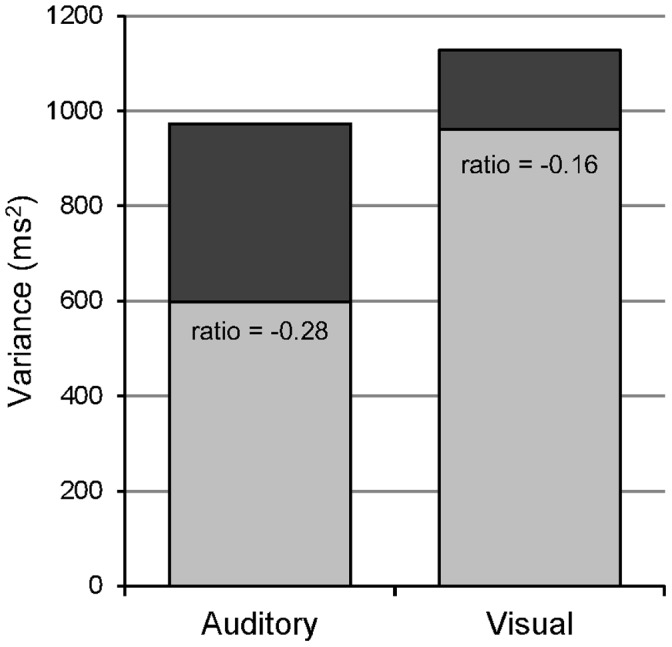
Effect of stimulus modality on decomposed performance variance. Cumulative mean estimates of variance. Timekeeper (light grey) and implementation variance (dark grey) are shown across the two stimulus conditions. Ratio values indicate the ACF which represents the relative amounts of variance from each source.

The variance in responses on the tapping tasks for each group and task modality was decomposed into timekeeper (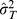) and implementation (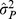) variance, as shown in Figure 3. Stimulus modality had a significant effect on timekeeper variance (F_(1,12)_ = 5.33, p<0.05, η^2^ = 0.31) with larger estimates obtained when tapping to visual stimuli (mean 907 ms^2^, S.E. 126.5) than to auditory stimuli (mean 611 ms^2^, S.E. 83.3). The effect of modality on implementation variance was not significant (F_(1,12)_ = 3.82, p = 0.07, η^2^ = 0.22), although the means (as illustrated in Figure 3) suggest that implementation variance was greater under auditory conditions (351 ms^2^, S.E. 55.8) compared to visual conditions (172 ms^2^, S.E. 26.7). A post hoc analysis collapsed across trials showed that this difference was significant statistically (t_(52)_ = 3.02, p<0.01). Neither timekeeper nor implementation variance was significantly affected by trial number nor were there significant interaction effects involving these variance components.

An assessment of the effect of modality on the ratio of timekeeper to implementation variance yielded a significant main effect (F_(1,12)_ = 5.32, p<0.05, η^2^ = 0.28). ACF was significantly lower in the auditory condition (mean = −0.28, S.E. 0.02) compared to the visual condition (mean = −0.16, S.E. 0.03) indicating that timekeeper variance is lower relative to implementation variance under auditory stimulation. The effect of trial and additional interaction effects were not significant statistically.

### Relationships between Motor Timing and Cognitive/Behavioural Measures

Predictive relationships between the timekeeper and implementation variance components and performance on psychometric measures of cognition and behaviour can help illuminate the relevance of timing deficits for developmental disorders such as dyslexia and ADHD on which measures of literacy and attention tap core symptoms. To evaluate these relationships, we calculated Pearson’s product moment correlations, using component measures of motor skill and psychometric performance to reduce the risk of Type 1 error associated with the large number of multiple comparisons that would arise from conducting pair-wise correlations between all of the measures in our task set. Summary measures of timing performance were created by averaging each of the timing variables (timekeeper variance, implementation variance and ACF) across the three trials within each modality. These composite values were considered appropriate because none of the variance components were influenced by trial number. When appropriate, variables were transformed to adhere to normality assumptions. The effects of outliers were also evaluated to ensure the validity of the correlation coefficients obtained [54]. The results of these analyses are shown in Table 2.

**Table 2:** Pearson’s product moment correlations and partial correlations.

	1.	2.	3.	4.	5.	6.	7.	8.	9.	10.	11.	12.
**1. Reading**	–	0.82**	−0.20	−0.14	−0.06	0.30	−0.04	0.43	−0.04	−0.53**	0.32	0.39
**2. Spelling**	.66**	–	−0.32	−0.14	0.12	0.02	−0.03	0.32	−0.05	−0.34	0.34	0.27
**3. ADHD-IA**	−.32	−.46*	–	0.80**	−0.07	−0.03	−0.07	−0.07	0.15	−0.04	−0.15	−0.03
**4. ADHD-HI**	−.38	−.38	.83**	–	0.23	0.08	−0.05	0.20	0.33	−0.09	−0.14	0.18
**5. Attention Control**	−.27	−.02	−.08	.18	–	−0.40	0.20	0.39	0.08	0.04	0.04	0.12
**6. Sustained Attention**	.28	−.10	−.03	.08	−.44	–	−0.05	0.29	0.50*	−0.48*	−0.21	0.65**
**7. Auditory TK variance**	−.09	−.08	−.07	−.10	.18	−.04	–	0.21	0.05	0.10	0.62**	0.08
**8. Visual TK variance**	.22	.05	−.09	.10	.33	.28	.18	–	0.09	−0.56**	0.20	0.74**
**9. Auditory IMP variance**	−.10	−.13	.16	.28	.04	.56*	−.01	.02	–	−0.14	−0.55**	0.25
**10. Visual IMP variance**	−.49*	−.24	−.03	−.07	.09	−.46*	.11	−.55*	−.17	–	0.03	−0.79**
**11. Auditory ACF ratio**	.16	.20	−.18	−.19	−.01	−.28	.66**	.10	−.59**	.13	–	0.04
**12. Visual ACF ratio**	.20	.05	−.05	.16	.06	.65**	.09	.75**	.32	−.77**	−.10	–

* p<0.05;

** p<0.01.

Pearson’s product moment correlations (top right) between psychometric variables of interest and motor timing performance, with partial correlations controlling for verbal and non-verbal reasoning (bottom left). TK = Timekeeper, IMP = Implementation, ACF = Autocorrelation function.

#### Associations with auditory timing

Auditory implementation variance was positively correlated with performance on the measure of sustained attention, with poorer attention associated with decreased implementation variance.

#### Associations with visual timing

Reading scores correlated positively with timekeeper variance and negatively with implementation variance. Sustained attention performance in the children was negatively associated with visual implementation variance, and positively with the visual ACF ratio measure. Thus, poorer sustained attention was associated with increased implementation variance overall, as well as relative to timekeeper variance.

A series of multiple regressions were performed to evaluate the importance of different timing variables as predictors of the cognitive/behavioural measures of interest. In the first analysis, reading ability was entered as the dependent variable, with the independent variables visual timekeeper variance and visual implementation variance entered in a fixed order, two-step model. This showed that visual implementation variance was a significant predictor (β = −0.53, t_(19)_ = −2.69, p<0.05) and alone accounted for 24% of the variance in reading accuracy scores (r^2^ = 0.28, F_(1,19)_ = 7.23, p<0.05). In contrast, timekeeper variance was not a significant predictor when entered alone at Step 1 (β = 0.43, t_(19)_ = 2.08, n.s.) or in the presence of implementation variance (β = 0.20, t_(18)_ = 0.83, n.s.). With both variables included at step 2, the equation remained significant but did not explain more variance in Reading performance beyond that contributed by implementation variance (Δr^2^ = 0.03, F_(1,19)_ = 3.9, p<0.05).

A second regression analysis evaluated the proportion of variance in sustained attention predicted by visual ACF, auditory implementation variance and visual implementation variance. Because implementation variance contributes to the ACF, only visual ACF was entered into the regression. The two predictors were entered step-wise into the equation in order of their strength of association with the dependent variable. The ACF in the visual modality was a significant predictor of sustained attention (β = 0.65, t_(19)_ = 3.76, p<0.01), accounting for 40% unique variance in this variable(r^2^ = 0.43, F_(1,19)_ = 14.16, p<0.01). The model remained significant with the inclusion of auditory implementation variance as a predictor (F_(2,18)_ = 10.78, p<0.01) and accounted for a further 9% of the variance in sustained attention (Δr^2^ = 0.12, β = 0.36, t_(18)_ = 2.16, p<0.05).

A final regression examined whether the dependent variable of visual implementation variance was more strongly related to reading performance or sustained attention, which were entered simultaneously as predictors. The model was significant (r^2^ = 0.43, F_(2,18)_ = 5.80, p<0.05) accounting for over 30% of the variance in visual implementation variance. Only reading performance was a significant unique predictor (β = −0.42, t_(18)_ = −2.17, p<0.05), in contrast to sustained attention (β = −0.36, t_(18)_ = −1.86, n.s.). These analyses suggest that reading ability is closely associated with implementation variance, whereas sustained attention is related more to the relative proportions of implementation variance and timekeeper variance.

## Discussion

Motor timing tasks provide clinically useful measures of temporal processing, which may help understand the mechanisms involved in developmental disorders such as ADHD and dyslexia for which deficits in implicit and explicit timing functions are a common feature [2], [5], [35], [36]. The increased recognition that risk factors for developmental disorders are expressed continuously in the population [8] underpins the need to establish the extent of overlap between disorder phenotypes on dimensions such as temporal processing. Previous studies of motor timing have presented stimuli via different stimulus modalities, however, with interval timing in ADHD assessed primarily using visual pacing stimuli and in developmental dyslexia with auditory stimuli. Here we provided a comprehensive assessment of such stimulus modality effects on paced motor timing performance in children, within the context of relationships with measures of literacy and attention variables. The addition of a variance decomposition method enabled us to evaluate sources of variability in finger tapping performance in children and to assess the contribution of these different underlying mechanisms to literacy ability and attention skills. The Wing and Kristofferson model [31] enabled separation of components that reflect the variability of both the ongoing timekeeping and the motor implementation of timing signals.

### Modality Dependent Effects

The data confirmed our hypothesis that finger tapping performance is strongly modulated by the modality of the pacing stimuli. Average IRIs in the auditory condition corresponded more closely with the target interval, compared to the visual condition where the intervals produced were significantly shorter and more asynchronous (Figure 2). In visual conditions, synchronisation of outputs with stimuli may be more difficult due to the lack of accurate temporal information available to a timekeeping mechanism [55], resulting in poorer precision of the clocking output for implementation [41]. Previous research has suggested that under such conditions participants may select and implement a response strategy irrespective of available information, for example asynchronies between stimuli and responses or feedback from internal timekeeping mechanisms [41], [42]. This response strategy hypothesis is supported by converging evidence from behavioural and brain imaging experiments. Errors in timing performance are typically neither noticed nor corrected under visually paced conditions, yet such errors are corrected sufficiently when performance is paced with auditory stimuli [41], [56]. Furthermore, neural areas engaged in updating of motor responses and recalibrating sensory-motor coupling are particularly active in auditory timing tasks but less so when timing is visually paced [42], [57].

Adopting stereotyped motor responses [41] would be expected to reduce implementation variance relative to timekeeper variance. Consistent with this explanation, we found that typically developing children had reduced implementation variance and increased timekeeper variance when pacing stimuli were presented visually. This pattern of result poses questions for the validity of using visually paced timing tasks alone in motor timing studies; the premise that timed motor outputs are always generated in concert with external pacing stimuli cannot be assumed. In sum, visually paced tasks may fail to adequately assess the internal timekeeping capacities that are of most interest when focusing on temporal processing and the putative difficulties thereof in relevant clinical populations.

In contrast to the results obtained with visual stimulation, participant responses in auditory conditions were characterised by lower estimates of timekeeper variance, coupled with higher estimates of implementation variance. This confirms that children, like adults [38], [40], [41], have relatively invariant output from timekeeper mechanisms when synchronising motor responses with auditory stimuli. This result reinforces evidence for the increased temporal precision of the auditory system compared to the visual system [38], [40] and highlights the important effects of task parameters in research of this kind. Studies employing visual, or bimodal auditory-visual stimuli [3], [16], [27] may underestimate the true capacities of central timing processes. Therefore, when considering questions of temporal processing in developmental populations, our results suggest that more confidence can be placed in results from auditory stimulated motor timing tasks than for similar visual paradigms [1], [14], [18], [24], [28].

### Relationships between Timing and Cognitive Variables

Timing tasks may explain unique variance in the underlying neuro-cognitive mechanisms of impairment in developmental disorders [2], [17], [18]. We therefore assessed the relationships between interval timing variables and the reading and attention variables that tap core behavioural symptoms upon which developmental dyslexia and ADHD are diagnosed. These two disorder phenotypes have been repeatedly studied with measures of interval timing, with visual tasks often used in investigations of ADHD [3], [16], [27], [58] and auditory tasks most frequent in studies of dyslexia [14], [23], [59].

The statistical associations between timing performance and indices of cognition and behaviour provide further support for the importance of the differences between timing assessed with visual and auditory pacing stimuli. Both sustained attention and reading ability were statistically associated with the relative contribution of implementation variance in finger tapping performance, and particularly for data derived from the visually paced task. Participants with low scores on these cognitive dimensions had relatively larger estimates of implementation variance on the visually paced task. Under visually paced conditions, timing mechanisms may lack precision, effects that are hypothesised to result from a combination of the inefficiency of the visual control mechanisms for generating internal rhythms [42], [60] and the poorer functional coupling of these mechanisms with the motor system [42], [57]. A stereotyped, motor-focused strategy has been suggested to be the most efficient approach to such tasks [41], [42] and would be predicted to result in reduced implementation variance. Our data suggest however, that children with lower scores on reading and attention measures do not consistently implement such a strategy on a visually paced task. Alternatively, it appears as if these children do not adequately account for imperfect timing signals. The specific demands of the visual timing task may elicit reallocation of cognitive resources to facilitate task completion [61], resulting in correlations between timing performance and reading and sustained attention variables similar to those that might be predicted for a measure of processing speed [62].

As applied in this study, the introduction of time series analysis to data obtained from motor timing tasks has helped to illuminate the potential links between individual variability in timing performance and in the cognitive dimensions that underlie highly prevalent developmental disorders. The Wing & Kristofferson model [31] has been applied previously to data obtained from other clinical populations with varying results [63]–[65], demonstrating the limitations of this approach for the analysis of data with very large IRIs, linear trends or negative variance estimates. Such parameters would not be unexpected in data derived from clinical groups, compared to that obtained from highly practiced individuals. The proportion of trials where the resulting data did not satisfy the assumptions of the model was comparatively modest in our sample compared to that reported in clinical groups [61], [62]. However, this proportion would be expected to increase in studies of children with developmental disorders. Rather than posing intractable problems for interpretation, however, such atypical data sets may provide additional information (for e.g., individual trends within time series [65]–[67]) useful for understanding the nature and extent of timing deficits in clinical or developmental populations [64], [68].

### Conclusions

Previous investigations of motor timing have reported associations between auditory paced timing tasks and measures of literacy, even in control populations [1], [24]. Our results do not provide strong evidence for this association. In contrast, they highlight the variable nature in the way auditory and visual tasks are processed behaviourally, as well as differences in the way that performance on temporal processing tasks correlates with cognitive constructs associated with highly prevalent disability phenotypes. They also highlight the methodological importance of assessing the construct of attention in temporal processing tasks [18], [61], particularly in clinical populations where attention difficulties often co-occur with the primary diagnostic symptoms [6], [7]. While the use of visual timing tasks may ultimately be useful for demonstrating the quality of processing difficulties experienced by children with attention deficits or reading difficulties, such measures may not adequately assess the timekeeping capability of central neural mechanisms. Our evidence suggests that central timekeeping mechanism(s) may be more accurately assessed with auditory paced tasks.
